# Pediatric Anthropometric Measurements of Amazonian Peruvian Children

**DOI:** 10.51894/001c.143938

**Published:** 2025-09-30

**Authors:** Brittany Bush

**Affiliations:** 1 College of Osteopathic Medicine Michigan State University

## 24

### INTRODUCTION

The World Health Organization (WHO) has developed a set of pediatric charts to monitor children’s growth and nutritional status around the world. However, there is controversy whether these charts accurately reflect local populations in Latin America because the data used by the WHO included only one locality in Brazil.

### HYPOTHESIS

The objective of the research is to assess the overall accuracy of the WHO growth charts for Peruvian children living in the Amazon. Further, it is hypothesized that pediatric charts for children, 3 moths to 5 years of age, work better for female than for male children.

### METHODS

Anthropometric data were gathered from the Amazonian region of Peru over a 3-year period with a total sample of 180 cases of children up to 5 years old. Measurements included weight, length or height, head circumference, mid-upper arm circumference, triceps skinfold, and subscapular skinfold. Along with these measurements demographic information about nationality, sex, dwelling, school attendance, and history of breast feeding were gathered. A focus was placed on the height and weight measurements for analysis. Bland-Altman plots were made to assess the degree of agreement of actual measured values and expected values according to the WHO standards. Also, the percentage of children outside of the WHO normal range was calculated.

### RESULTS

It was found that children up to age 5 in the Amazonian regions of Peru were both underweight and under height/length compared to the WHO values. The female bias was 0.56 kg underweight and 3.10 cm under the height estimate. The male bias was 0.80 kg underweight and 3.70 cm under the height estimate. According to WHO development standards, 3.5% of children are either moderate or severely underweight. In terms of height, 18.2% of children would be considered moderate to severely stunted.

### CONCLUSION

The analysis supports our hypothesis that the female WHO data is more representative of female Peruvian children. However, both genders show an overall bias in both height and weight indicating there is a need to develop appropriate charts or adjust measurements to reflect the characteristics of children in the Peruvian Amazon.

